# Insights and Advancements in Periodontal Tissue Engineering and Bone Regeneration

**DOI:** 10.3390/medicina60050773

**Published:** 2024-05-07

**Authors:** Angela Angjelova, Elena Jovanova, Alessandro Polizzi, Marco Annunziata, Ludovica Laganà, Simona Santonocito, Gaetano Isola

**Affiliations:** 1 University Dental Clinical Center St. Pantelejmon, Skopje, Faculty of Dentistry, Ss. Cyril and Methodius University in Skopje, 1000 Skopje, North Macedonia; angela.angjelova@students.stomfak.ukim.mk (A.A.); elena.jovanova@students.stomfak.ukim.mk (E.J.); 2 Department of General Surgery and Surgical-Medical Specialties, School of Dentistry, University of Catania, 95124 Catania, Italy; ludovicalagana99@gmail.com (L.L.); simonasantonocito.93@gmail.com (S.S.); 3 Multidisciplinary Department of Medical-Surgical and Dental Specialties, University of Campania Luigi Vanvitelli, Via L. De Crecchio, 6, 80138 Naples, Italy; marco.annunziata@unicampania.it

**Keywords:** tissue engineering, bone regeneration, stem cell therapy, growth factors, periodontal regeneration, biomaterials, periodontitis, dentistry

## Abstract

The regeneration of periodontal bone defects continues to be an essential therapeutic concern in dental biomaterials. Numerous biomaterials have been utilized in this sector so far. However, the immune response and vascularity in defect regions may be disregarded when evaluating the effectiveness of biomaterials for bone repair. Among several regenerative treatments, the most recent technique of in situ tissue engineering stands out for its ability to replicate endogenous restorative processes by combining scaffold with particular growth factors. Regenerative medicine solutions that combine biomaterials/scaffolds, cells, and bioactive substances have attracted significant interest, particularly for bone repair and regeneration. Dental stem cells (DSCs) share the same progenitor and immunomodulatory properties as other types of MSCs, and because they are easily isolable, they are regarded as desirable therapeutic agents in regenerative dentistry. Recent research has demonstrated that DSCs sown on newly designed synthetic bio-material scaffolds preserve their proliferative capacity while exhibiting increased differentiation and immuno-suppressive capabilities. As researchers discovered how short peptide sequences modify the adhesion and proliferative capacities of scaffolds by activating or inhibiting conventional osteogenic pathways, the scaffolds became more effective at priming MSCs. In this review, the many components of tissue engineering applied to bone engineering will be examined, and the impact of biomaterials on periodontal regeneration and bone cellular biology/molecular genetics will be addressed and updated.

## 1. Introduction

Periodontitis is a chronic inflammatory condition characterized by the progressive degradation of the periodontal tissue responsible for tooth support. In the absence of appropriate treatment, it has the potential to induce tooth mobility and tooth loss [1,2], through mechanisms that involve several mediators [3]. Conventional nonsurgical periodontal treatment consistently diminishes inflammation levels and probing pocket depths while augmenting the attachment level and inducing an apical displacement of the gingiva. It is crucial to emphasize that conventional nonsurgical periodontal treatment is constrained by factors, including the sustained management challenges associated with deep periodontal pockets and the potential of disease relapse [4]. Furthermore, this periodontal treatment gives rise to residual pockets, thereby adversely impacting the prognosis of the tooth. The prevention of these results can be achieved through the implementation of advanced periodontal regenerative approaches, which aim to re-establish the compromised periodontal tissues [5].

In this regard, the state of the art is that the microenvironment that initiates the generation of tissues supporting teeth is not maintained following tooth development, thereby posing challenges in the restoration of the lost periodontal ligament after reaching maturity [6]. As an advanced technology, the in situ tissue engineering approach, employing biomaterials for controlled delivery adaptable to recapitulating this environment, is considered a promising technology for the treatment of periodontal bone defects [7]. To achieve this objective, numerous biomaterials were established, significantly influencing oral tissue. They function as three-dimensional scaffolds featuring surfaces that enhance cellular adhesion, proliferation, and differentiation, thereby establishing a supportive environment for regeneration. Moreover, biomaterials can come in immediate contact with the living tissue, without developing any adverse immune reaction [8].

Regeneration of periodontal tissues plays a crucial role in this broad field, involving the restoration of cementum, periodontal ligament (PDL), and the alveolar bone encasing teeth. To develop successful strategies for periodontal and tissue engineering therapies, it’s essential to merge knowledge from various disciplines like molecular and cellular biology, medicine, and material science in an interdisciplinary manner [9]. The organically complex structure of bone possesses a highly specialized organic–inorganic architecture that includes an inorganic phase contributing to its hardness, a mineral phase consisting of carbonated apatite, and additional noncollagen proteins that create a microenvironment conducive to sustaining cellular functions [10]. In this context of periodontal regeneration, tissue engineering, and regenerative medicine have undergone substantial advancement over the years, originating from the early concept of guided tissue regeneration (GTR) [9].

The GTR concept involves employing barrier membranes with cell-occlusive properties and this strategy aims to specifically hinder the migration of epithelial and fibroblastic cells, while simultaneously facilitating the replenishment of damaged areas by encouraging the migration of cells originating from the periodontal ligament, bone, and cementum [11]. Guided bone regeneration (GBR) was devised to regenerate osseous defects by applying the principles of GTR, consequently, GBR integrates the application of barrier membranes to physically prevent the infiltration of soft tissue into the defect site, facilitating repopulation with osteogenic progenitor cells [12]. The components of treatment strategies in tissue engineering comprise signaling molecules/growth factors, scaffolds, and cells, with particular attention focused on promoting osteogenesis, angiogenesis, and controlling inflammation [13]. Following the initial creation of GTR and GBR, notable progress has occurred in the integration of scaffolds, biology, and gene therapy. The objective is to influence the healing response to enhance and promote regeneration [14]. However, these methodologies remain unreliable in their ability to effectively regenerate intact periodontal tissue affected by severe periodontitis, therefore the stem cell-based approaches in tissue engineering and regenerative medicine hold promise as treatment strategies for periodontal tissue regeneration (PTR) [15].

This article commences with an exploration of periodontal regeneration and bone cellular biology, alongside an examination of strategies for periodontal tissue regeneration, which encompass techniques such as GTR and tissue engineering. Following this, the focus shifts to the discussion of biomaterials, with an emphasis on the advancement of sophisticated scaffolding biomaterials. Furthermore, a summary is provided regarding the application of growth factors on periodontal cells and the progress in cellular and bone regenerative molecules for regenerating periodontal tissues, specifically targeting the periodontal ligament (PDL) and alveolar bone. Lastly, we present future perspectives on the utilization of bio-inspired materials for the reconstruction of bone defects, serving as a beacon for future advancements in this field.

## 2. Periodontal Regeneration and Bone Cellular Biology

Periodontal regeneration refers to the renewal of tissue supporting the tooth, encompassing cementum, periodontal ligament (PDL), and alveolar bone. The primary objective in periodontal regeneration is the formation of new cementum, accompanied by the integration of PDL into the alveolar bone. Throughout the healing phase, in the periodontal treatment, epithelial cells, known for their rapid migratory capability, establish the long junctional epithelium. This particular mode of the healing process delays the regeneration of other structural components within the apparatus [9].

Osteoblasts, osteocytes, and bone lining cells originate from osteogenic cell lineage, which arises from mesenchymal cells located within the bone marrow [16]. Mature osteocytes, situated within the bone matrix, aid in releasing calcium from the matrix and exchanging it with bodily fluids in response to systemic needs. Meanwhile, osteoclasts, originating from hematopoietic stem cells and specialized in bone resorption, are pivotal in the degradation of mineralized bone. They greatly contribute to the normal growth and development of bone, as well as the lifelong maintenance of bone integrity [17]. The ongoing osseous remodeling process involves the incessant removal of mature bone by osteoclasts, while simultaneously, osteoblasts are engaged in the concurrent formation of new bone tissue. In the resorptive phase, osteoclasts collaboratively operate to eliminate both mineral and organic constituents of the bone matrix [18]. Recent technological progress, such as intravital imaging in bone research, has provided fresh insights into osteoclast biology. Studies have revealed a different fate for osteoclasts, wherein they divide into daughter cells called osteomorphs. Interestingly, these newly formed entities are seen circulating in the bloodstream and settling in the bone marrow, where they later rejoin other osteoclasts on the bone surface—a process termed osteoclast recycling [19].

On the other hand, the initiation of new bone formation, a biphasic procedure, commences following a brief reversal phase, initiating with the deposition of osteoid. The organic matrix, primarily composed of type 1 collagen and various other elements, undergoes subsequent mineralization over an approximately 20-day duration [17].

In pathological conditions, an alteration in the crucial equilibrium necessary for functional bone turnover leads to deleterious osteolytic mechanisms. In inflammatory periodontal disease, both microbial and host-derived factors play a role in the processes of bone resorption and remodeling [20,21]. The process of alveolar bone remodeling closely resembles that of general bone remodeling. Nevertheless, resorption occurs asynchronously, resulting in focal loss of periodontal ligament attachment. In the context of tooth movement, force distribution ensures a compensatory mechanism wherein bone loss from resorption on one aspect of the alveolus is counteracted by bone deposition on the opposing aspect. This equilibrium, in conjunction with the ongoing cementum deposition, serves to sustain a relatively consistent association between the root and the dental alveolus [20].

**Figure 1:**
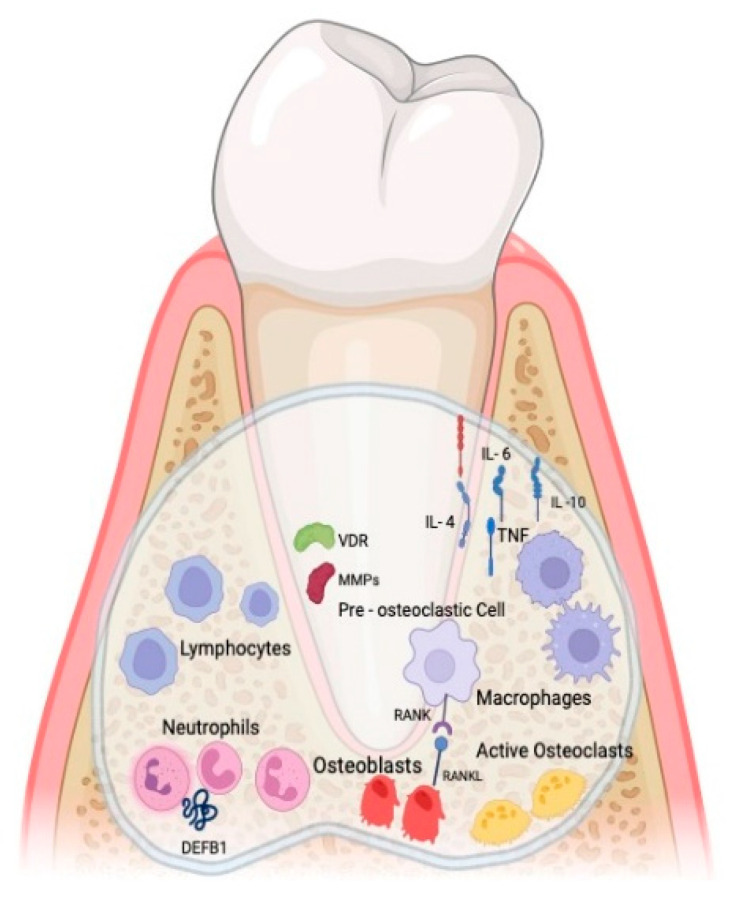
Schematic illustration of lymphocytes, neutrophils, osteoblasts, macrophages, osteoclasts, and proinflammatory cytokines identified in the process of bone resorption. Created by BioRender.com.

Bacteria induce tissue damage indirectly by amplifying the host’s immune response. Recent advancements in the acquired immune responses, specifically concerning B-lymphocytes, T-lymphocytes, and inflammatory mediators in the context of periodontal disease progression, have been thoroughly examined. Several proinflammatory cytokines and growth factors, notably IL-1 and tumor necrosis factor-alpha (TNF-α), are identified in association with bone resorption (Figure 1). The intricate balance of bone remodeling hinges on the equilibrium between bone formation and bone resorption. The receptor activator of nuclear factor-kappa B (RANK) and its ligand RANK-L, both belonging to the tumor necrosis factor receptor superfamily, directly influence osteoclast precursor differentiation and the activation and survival of osteoclasts. Research indicates that proinflammatory cytokines like IL-1 and TNF-α, significant contributors to periodontal bone loss, regulate the expression of RANK-L and osteoprotegerin. Moreover, T-cells express RANK-L, which, upon binding to RANK on the cell membrane of osteoclast progenitors, preosteoclasts, and osteoclasts, promotes both cell differentiation and activation within the osteoclast lineage. Consequently, the deviation in bone homeostasis toward bone resorption in periodontitis is likely orchestrated by proinflammatory cytokines that control the expression of RANK-L on both mesenchymal cells and specific activated T-cells [22,23,24,25,26].

The dysregulation of RANK-L and osteoprotegerin (OPG) expression during inflammatory states, characterized by elevated RANK-L and diminished OPG activity, leads to heightened osteoclast genesis and increased bone resorption. Furthermore, while the immune response triggered by bacteria and their byproducts serves as a defensive mechanism, it can also cause varying degrees of harm to the organism. In cases of inflammation around alveolar bone tissue, there is a notable correlation between the inflammatory response mediators and bone tissue metabolism. Conversely, cytokines play a crucial role in coordinating the activities of inflammatory cells to facilitate repair. Some studies involving animals lacking specific receptors and cytokines provide substantiation for the correlation between the expression of anti- and proinflammatory cytokines and their association with the induction of osteoclast genesis [27].

Recent research in periodontal and bone regeneration strives to create therapeutic approaches that can recruit and guide host cells in a specific time and space, thereby enhancing regeneration and healing. Besides identifying suitable biologics for regenerative interventions, it is imperative to ascertain the optimal dosages and delivery modalities. Moreover, substantial progress has been achieved since the inception of guided tissue regeneration (GTR) and guided bone regeneration (GBR), particularly in utilizing biologics, scaffolds, and cell-based therapies to modulate the healing process, thereby promoting regenerative outcomes [28].

## 3. Biomaterials and the Purpose of Using Scaffold Design for Periodontal Regeneration

### 3.1. The Strategies of Periodontal Regeneration

Two methodologies for the regeneration of periodontal tissues can be recognized: guided tissue regeneration (GTR) and tissue engineering. GTR, extensively employed in periodontium regeneration, constitutes a regenerative surgical technique involving the elevation of mucogingival flaps, scaling of the root surfaces, and the temporary placement of barrier membranes beneath the gingiva. The underlying biological rationale of GTR is centered on impeding the apical progression of epithelium towards the root surface, achieved through the utilization of barrier membranes. Consequently, this obstruction facilitates the formation of periodontal ligament (PDL) tissues and alveolar bone by enabling the proliferation of PDL cells and osteoblasts [29,30]. The use of Millipore filters in guided tissue regeneration (GTR) for treating periodontal conditions was noted to lead to the formation of a new attachment by the periodontal ligament, without the presence of long junctional epithelium or ankylosis. Despite the apparent success of GTR procedures, some studies have suggested the potential risk of root resorption and ankylosis. Moreover, when the regenerative potential of the periodontal ligament is significantly reduced due to prolonged periodontitis, achieving coordinated regeneration of various periodontal tissues becomes challenging [31].

As an innovative resolution to surmount challenges, recent investigations have explored tissue engineering methodologies for the regeneration of periodontal tissues. Classifiable by the utilization of biomaterials, the tissue engineering strategy for periodontal regeneration can be classified into scaffold-free and scaffold-based methodologies. In the scaffold-free approach, cells are transplanted into a defective area without the incorporation of a cell carrier. Conversely, the scaffold-based approach involves the utilization of stem/progenitor cells, scaffolds, and bioactive molecules to fabricate biomimetic systems that facilitate the initiation of new tissue formation. When combined with cells and/or bioactive agents, diverse biomaterial scaffolds can be utilized in conjunction with GTR. Recently, more sophisticated scaffold systems have been developed to facilitate the orchestrated regeneration of the periodontium. These scaffolds are specifically designed to administer bioactive signals conducive to periodontal tissue regeneration while undergoing controlled degradation, subsequently being substituted by newly formed tissues [3,31,32].

### 3.2. Biomaterials for Guided Tissue Regeneration (GTR)

**Figure 2:**
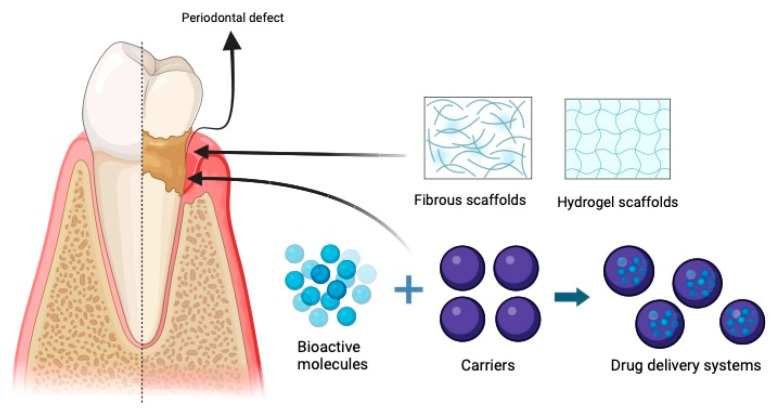
Schematic illustration of the anatomy of periodontal tissues, periodontal defect, scaffolds of tissue engineering, and drug delivery system. Created by BioRender.com.

GTR, or guided tissue regeneration, denotes the process of restoring periodontal tissue by employing a barrier membrane that obstructs the interface between epithelial and alveolar bone/periodontal ligament (PDL) tissues. In this procedure, a membrane is introduced to the operative site to impede the migration of connective and epithelial tissue across the operative area [33,34]. Using a barrier membrane serves as a technique to prevent epithelial cell ingress, facilitating the regeneration of periodontal tissues in a manner that maintains the original structure and functionality, rather than restoration with junctional epithelium. During the obstructive phase, several cell types including cementoblasts, osteoblasts, osteoclasts, and mesenchymal cells from the periodontal ligament become activated, aiding in the reconstruction of their respective deficient tissues [35]. A barrier membrane must meet specific criteria, including tissue adhesion without mobility, prevention of soft tissue from growing in the defect, ease of application, maintenance of a defined place, and biocompatibility. Presently, membranes are categorized into two types: nonresorbable and resorbable [36] (Figure 2).

Expanded polytetrafluoroethylene (e-PTFE) emerged as the standard for bone regeneration by the early 1990s. It is created by subjecting PTFE to high tensile stress. However, a drawback of e-PTFE is the challenging removal of the membrane necessitating extensive releasing incisions. To address this issue, a high-density PTFE membrane (d-PTFE) with a pore size of less than 0.3 microns was introduced in 1993. The removal of d-PTFE is simplified as there is no tissue ingrowth into the surface structure. Nonetheless, a disadvantage is the membrane’s tendency to collapse towards the defect [37]. Nonresorbable presents several disadvantages, including the requirement for a second surgical procedure for removal, early exposure to the oral environment, and the risk of wound dehiscence. In response to these challenges, resorbable membranes have been developed. At present, there are two categories of resorbable membranes: those composed of polymers called synthetic membranes and those derived from collagen obtained from animal sources or called natural membranes [37].

Natural membranes primarily comprise collagen derived from human skin, bovine Achilles tendon, or porcine skin. Collagen demonstrates a range of biological functions, including hemostasis, attraction of periodontal ligament and gingival fibroblast cells, augmentation of soft tissue, and biocompatibility and biodegradability. However, in vivo, collagen-based membranes often exhibit suboptimal performance due to their degradation over time. Von Arx and Buser emphasized the rapid degradation of non-cross-linked collagen membranes as a beneficial aspect in horizontal ridge augmentation procedures. This rapid degradation allows for spontaneous re-epithelialization within 2 to 4 weeks, eliminating the necessity for secondary surgery to remove the membrane [35,36,38].

Polymeric membranes are constructed from polyesters like polyglycolic acid (PGA), polylactic acid (PLA), or their copolymers. These materials possess characteristics of biocompatibility, biodegradability, and ease of manipulation. Despite their typical biodegradability, the application of these polymeric membranes has been linked to inflammatory responses within the body. Around the implanted membrane, one may observe either fibrous encapsulation or infiltration by inflammatory cells. The combination of natural and synthetic polymers merges the bioactive properties inherent in natural materials with the superior mechanical characteristics of synthetic materials. Additionally, GTR membranes have been utilized as vehicles for drug delivery to enhance tissue regeneration [3,33,35,36,39].

The advancement of barrier membrane technology has led to a growing interest in functional membranes in recent years. Three primary approaches to construction have emerged: (1) the incorporation of antibacterial agents, (2) the incorporation of bioactive factors, and (3) the utilization of a multilayered architecture [40].

The incorporation of antibacterial drugs into GTR membranes serves to impede local infection and inflammation, thereby facilitating the formation of PDL tissue. Electrospinning represents a widely used technique for producing drug-incorporated biodegradable membranes. The resulting fibers exhibit a large surface area-to-volume ratio and a three-dimensional network structure, enhancing cell adhesion, porosity, and proliferation [41]. Furthermore, multilayered GTR membranes with distinct functionalities in each layer have been devised to amplify the process of periodontal tissue regeneration (Table 1) [3,33,35,36,39].

Advancements in synthetic materials and polymers should be incorporated into the design of barrier membranes to improve future material development. It’s crucial to integrate antimicrobial agents and properties, as well as utilize nanomaterials technology, to minimize potential complications during regenerative therapy and maximize treatment effectiveness [42].

### 3.3. In Situ Tissue Engineering Biomaterials

Tissue engineering represents an interdisciplinary domain that has evolved from the evolution of biomaterials designed to restore or uphold the function of damaged tissues or organs. The concept of tissue engineering was introduced by Langer and Vacanti as a prospective technique for regenerating lost tissues. In periodontics, tissue engineering strategies primarily concentrate on the regeneration of oral soft tissues and alveolar bone. These approaches incorporate three essential components to augment tissue regeneration: progenitor cells, a scaffold or supportive matrix, and signaling molecules [43]. In the context of regenerating dental tissue, scaffolds and biomaterials play crucial roles, where they function as sites for the adherence of regenerative cells originating from adjacent tissues, provide a framework for guiding tissue regeneration, serve as a reservoir of implantable odontogenic cells capable of differentiating into specific cell types, and act as carriers for bioactive molecules, particularly growth factors that enhance the regenerative potential [44].

Categorized according to their biocompatibility, biomaterials for in situ tissue engineering are classified as bioactive, biotolerant, biodegradable, and bioinert [45].

Bioactive materials have the potential to stimulate osteogenesis by eliciting a biological response, typically through the establishment of chemical bonds. Examples include bioactive glasses, hydroxyapatite, and β-tricalcium phosphate. Biotolerant materials, including polymers and most metals, are generally well accepted by the host but remain physically separated from host tissue by fibrous tissue formation. Biodegradable materials, such as polyglycolic acid polymers, calcium phosphates ceramics, and certain biodegradable metals, undergo dissolution upon exposure to bodily fluids. Bioinert materials, like titanium, demonstrate stability within the human body and can, under specific circumstances, form both structural and functional connections with adjacent bone (Table 2) [44,46,47].

In tissue engineering, scaffolds play a vital role by maintaining space, storing growth factors, and aiding cell attachment and proliferation when applied to bone defects. Adding growth factors or stem cells enhances the osteoinductive and osteogenic properties of these scaffolds. The porous nature of the scaffolds offers benefits for loading growth factors, facilitating cell attachment, and promoting growth and proliferation [48].

Nonetheless, the majority of materials employed in periodontal regeneration are conventionally utilized in other areas of regenerative research and are incapable of replicating the structures found in natural periodontal tissues. As a result, it is imperative to develop innovative and bio-inspired materials engineered to closely emulate the micro- and nanoscale architecture of periodontal tissues, which are essential for achieving effective regeneration of functional periodontal tissues [3]. In the past few years, significant advancements have been made in the development of hydrogel scaffolds using diverse biocompatible materials, with specific functionalities aimed at applications in bone tissue engineering. These hydrogel scaffolds have been successfully demonstrated to be implantable within bone tissues through various modalities including intravenous injection, subcutaneous implantation, in situ injection, or surgical placement, demonstrating notable therapeutic efficacy in addressing bone-related disorders [49].

## 4. Controlled Delivery Systems for the Regeneration of Periodontal Tissues

### 4.1. Scaffold in Combination with Growth Factors

Growth factors, proteins capable of exerting influence either locally or systemically, can affect cell growth and function through multiple mechanisms, including inducing chemotaxis, proliferation, differentiation, extracellular matrix synthesis, and angiogenesis. The use of growth factors in tissue repair aims to achieve regeneration by employing biomimetic processes that mimic those occurring during embryonic and postnatal development. While these molecular mediators exhibit diverse biological functions, the selection of specific factors for regenerative therapy is based on their crucial roles in periodontal tissue development and wound healing [9,50]. Given the crucial function of growth factors (GFs) in regulating cellular activities and their capacity to directly facilitate and manipulate tissue regeneration, a broad spectrum of GFs has been investigated and examined for therapeutic purposes, including applications in bone regeneration (Table 3) [51].

Due to the fundamental role of GFs in modulating cellular functions and their direct capacity to stimulate and facilitate tissue regeneration, a diverse array of GFs have undergone extensive study and evaluation for therapeutic applications, including those related to bone regeneration. Fibroblast growth factors (FGFs), vascular endothelial growth factors (VEGFs), insulin-like growth factors (IGFs), transforming growth factors-β (TGF-β), platelet-derived growth factors (PDGFs), and bone morphogenic proteins (BMPs) constitute the principal groups of GFs associated with bone regeneration. GFs such as PDGFs, BMPs, IGFs, TGF-β, and VEGFs exhibit considerable potential in bone healing by modulating various cellular behaviors, including recruitment, migration, adhesion, proliferation, and differentiation. A combination of angiogenic factors (VEGF), cell recruiting factors (PDGF), and osteogenic factors (BMPs) has been devised and demonstrated to exert a synergistic effect, proving more advantages for bone repair compared with the administration of any single GF in isolation [45,46,47]. The implementation of growth factors (GFs) via scaffold-based approaches aims to orchestrate cellular reactions by bridging the communication between cell signaling and the dynamics of bone injury recovery. Tissue engineering scaffolds are anticipated to not only impede ectopic bone formation by facilitating swift infiltration of host cells from the periphery to the core of the scaffold but also to demonstrate minimal immunogenic and antigenic reactions [52,53,54].

#### 4.1.1. Platelet-Derived Growth Factor (PDGF)

Platelet-derived growth factor (PDGF) is a naturally occurring protein abundantly present in the bone matrix. It manifests as dimmers composed of A, B, and C polypeptide chains, and four distinct isomeric forms have been recognized: PDGF-AA, PDGF-AB, PDGF-BB, and PDGF-CC. The signaling through PDGF receptors has been documented to exert a significant influence on the control of proliferation and migration of various cells, including osteoblasts and fibroblasts [55]. Recombinant human platelet-derived growth factor-BB stands out as one of the extensively investigated GD applied in the field of periodontal tissue engineering. Since its introduction in the late 1980s, rh-PDGF-BB has been made commercially accessible in the form of a Growth-Factor-Enhanced Matrix (GEM). This product incorporates β-tricalcium phosphate as a scaffold [50,51,55]. Residing within the ζ-granules of platelets and discharged upon activation, PDGF has been demonstrated to stimulate DNA synthesis, chemotaxis, and the synthesis of collagen and glycosaminoglycans in fibroblasts [56,57,58]. Laboratory analyses conducted in vitro have shown that PDGF significantly enhances the expression of stem cell markers and promotes the proliferation of mesenchymal stem cells (MSCs) isolated from PDL tissues [59].

#### 4.1.2. Fibroblast-Derived Growth Factor (FGF)

Fibroblast growth factor-2, alternatively recognized as basic fibroblast growth factor, exhibits potent mitogenic characteristics and plays crucial roles in processes such as wound healing, granulation tissue formation, angiogenesis, and tissue remodeling [60,61,62]. Heparin and heparin sulfates are crucial in enhancing the bioactivity of FGF. The association of FGF-2 with heparin significantly decreases the degradation of FGF-2 and greatly enhances its mitogenic activity. Additionally, heparin sulfate proteoglycans act as cofactors in the interactions between FGF2 and FGF receptors, facilitating the activation of downstream signaling pathways [55,56,57]. In general, clinical data substantiates the safety and effectiveness of employing FGF-2 in the treatment of intraosseous defects in periodontal conditions. However, further clinical trials are imperative to substantiate the application of this growth factor in the realms of bone regeneration [63,64].

#### 4.1.3. Transforming β Growth Factor (TGF-β)

Bone morphogenetic proteins (BMPs) represent the most extensive subset within the transforming growth factor-β. (TGF-β) family. Presently, fourteen BMPs have been characterized, among which BMP 2, 4, 5, 6,7, and 9 exhibit osteoinductive capabilities. Among these, BMP-2 and BMP-7 have been subject to comprehensive investigation in the context of periodontal tissue engineering objectives [59,60,61]. While the application of BMPs in the regeneration of periodontal tissues has demonstrated efficacy in addressing intrabony and furcation defects, adverse events such as ankylosis and root resorption have been documented. Consequently, BMPs are presently recommended specifically for implant site preparation in the context of ridge preservation and sinus floor augmentation procedures [9].

Growth and differentiation factor (GDF)-5 is also a member of the TGF-β superfamily of proteins, exhibiting a structural similarity to BMPs. GDF-5 is actively involved in wound healing and periodontal regeneration processes through the modulation of extracellular matrix metabolism [62,63].

#### 4.1.4. Insulin-like Growth Factor (IGF)

The coadministration of insulin growth factor one (IGF-1) and PDGF has previously demonstrated an augmentation of the healing process for soft tissue wounds. Some initial findings imply that in vivo utilization of the PDGF and IGF-1 combination may promote the regeneration of periodontal structures. In several other investigations, it has been demonstrated that PDGF-BB alone significantly promotes periodontal regeneration. However, IGF-1 alone does not exert any discernible effect on periodontal wound healing in a nonhuman primate model [50,64].

### 4.2. Novel Findings in Scaffolds Incorporating Growth Factors for Enhanced Periodontal Regeneration

A recent study investigated the development of an in-situ tissue engineering scaffold (iTE-scaffold) using the coaxial electrospinning technique to sequentially deliver bFGF and BMP-2 [65,66,67,68]. The iTE-scaffold was found to effectively enhance the angiogenesis of periodontal ligament stem cells (PDLSCs) and induce macrophage polarization into the prohealing M2 phenotype, thereby modulating inflammation [7].

Enamel matrix derivative (EMD) is a protein believed to stimulate cell proliferation, migration, and differentiation within periodontal tissues when incorporated into a suitable scaffold, and it could potentially exhibit increased effectiveness [69,70]. In the group treated with EMD, there was a notable increase in new bone formation [71].

Moreover, the human amniotic membrane (hAM) has been demonstrated to possess a high concentration of growth factors, including epithelial growth factor (EGF), bFGF, TGF-α and β, VEGF, and hepatocyte growth factor. These growth factors are associated with periodontal regeneration due to their ability to stimulate the growth of endogenous progenitor cells. While the specific effectiveness of hAM in promoting periodontal regeneration remains uncertain, it has been identified as a promising scaffold for periodontal regenerative therapy [72].

## 5. Cell-Based Tissue Engineering

Regenerative medicine encompasses the substitution or rejuvenation of human cells, tissues, or organs. Langer and Vacanti propose that tissue engineering comprises a triad, which includes stem/progenitor cells, scaffolds supporting cell growth, and essential growth factors [65,66,67]. Termed the paracrine effect, stem cells demonstrate an exceptional ability to release cytokines locally, thus promoting regenerative processes and influencing the balance between tissue regeneration and degeneration. Mesenchymal stem cells (MSCs) can be obtained from various sources including bone marrow, peripheral blood, umbilical cord blood, adult connective tissue, placenta, amniotic membrane, and dental tissues. However, MSCs derived from dental tissues represent readily accessible multipotent cells [68,69].

**Figure 3:**
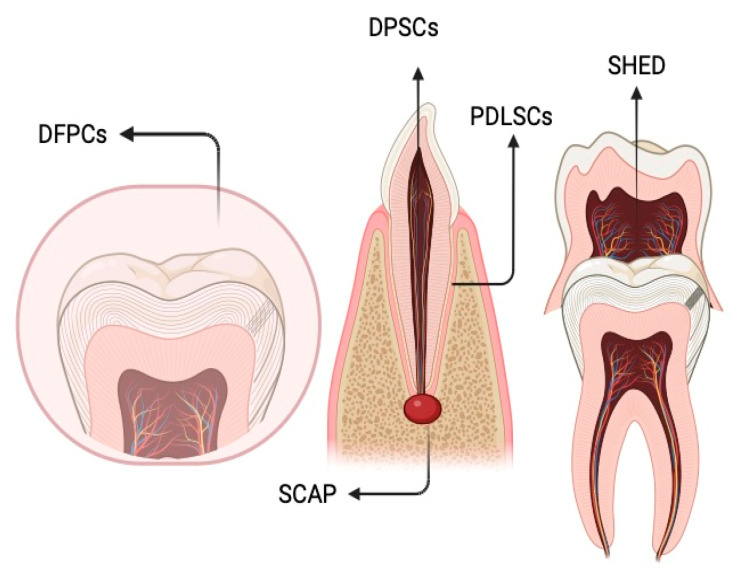
Schematic image of human DSCs. Abbreviations: DFPCs—dental follicle progenitor cells; PDLSCs—periodontal ligament stem cells; SCAP—stem cells from apical papilla; DPSCs—dental pulp stem cells; SHED—stem cells from exfoliated deciduous teeth. Created by BioRender.com.

Dental stem cells (DSCs) comprise populations resembling MSCs characterized by their ability for self-renewal and potential for multidifferentiation. Initially, dental pulp stem cells (DPSCs) were the first DSCs to be isolated and characterized. Subsequently, additional varieties of DSCs were identified, including stem cells from human exfoliated deciduous teeth (SHED), periodontal ligament stem cells (PDLSCs), dental follicle precursor cells (DFPCs), and stem cells from apical papilla (SCAP) [70,71,72]. These five categories of DSCs demonstrate remarkable multipotency, showcasing the capacity for differentiation into various cell lineages, including osteogenic, odontogenic, dentinogenic, cementogenic, adipogenic, myogenic, and neurogenic pathways [73,74] (Figure 3).

Within the framework of periodontal therapy, a prospective tissue engineering strategy for periodontal regeneration entails embedding progenitor cells within a premanufactured three-dimensional structure, which is then transplanted into the site of the defect. This approach addresses certain constraints linked to traditional regenerative procedures by facilitating the direct placement of growth factors and progenitor cells into the defect [32]. To achieve positive outcomes in periodontal tissue engineering, several essential components are required: (1) an ample supply of appropriate progenitor cells capable of differentiating into essential mature phenotypes for tissue formation, including osteoblasts, cementoblasts, and fibroblasts; (2) the necessary signals to regulate cellular differentiation and tissue regeneration; and (3) a supportive three-dimensional extracellular matrix scaffold that enables and maintains these processes [75,76,77,78]. Moreover, angiogenic signals play a crucial role in stimulating the formation of new vascular networks, serving as a vital foundation for tissue growth. Additionally, proper mechanical loading is indispensable for the establishment of a well-organized, functional PDL. Lastly, given the microbial presence in periodontal defect sites, strategies for infection control and managing the host response become imperative for optimal periodontal regeneration [79].

In recent years, the microsphere-based cell system has been recognized as an advantageous expansion platform due to its provision of a high surface-to-volume ratio, facilitating the large-scale cultivation of anchorage-dependent cells, such as MSCs. MS-based scaffolds can be categorized into two types: (1) MS-incorporated scaffolds, where MSs are integrated into bulk scaffolds to enhance their biological functionalities, and (2) MS-leached scaffolds, where MSs serve to generate porous scaffolds These scaffold varieties have been extensively applied in tissue engineering for the regeneration of cartilage, dental, neural, bone, and skin tissues. Furthermore, the MS–cell system shows potential as an effective strategy for tissue repair and therapeutic intervention in various disorders [80,81].

### 5.1. Bone-Marrow-Derived MSCs (BMSCs) for Periodontal Regeneration

Multipotent progenitor cells known as bone-marrow-derived mesenchymal stem cells (BMSCs) are found in the adult bone marrow. These cells exhibit a replicative capacity, allowing them to differentiate into various connective tissue cells. While BMSCs can be isolated from the iliac crest, they are also obtainable from orofacial bones [80,81]. Human BMSCs, specifically from the maxilla and mandible, can be isolated from bone marrow aspirated during dental procedures such as dental implantation, third molar extraction, orthodontic osteotomy, or cyst extirpation [82,83,84,85]. The autotransplantation of MSCs derived from bone marrow has demonstrated the ability to stimulate regeneration up to 20% in both cementum and alveolar bone. Additionally, a methodology involving genetic engineering of bone-marrow-derived MSCs to express a green fluorescent protein (GFP) revealed that four weeks post transplantation, the periodontal defects exhibited nearly complete regeneration, featuring GFP-positive cementoblasts, osteoblasts, osteocytes, and fibroblasts within the regenerated periodontal tissue [32]. However, studies have shown that the quantity obtained from bone marrow in the iliac crest is greater than that from orofacial bone marrow [86,87,88]. In conclusion, the authors propose that, when utilizing BMSCs in clinical trials, it is imperative to establish a secure cell expansion method and a more dependable protocol [82,89].

### 5.2. Adipose-Derived Cells

The utilization of adipose tissue as a source of stem cells has been investigated, representing an additional extraoral and nondental reservoir of MSCs with potential relevance in periodontal tissue engineering. Adipose-derived stem cells (ADSCs) can be acquired through lipectomy or lipoaspiration from regions such as the chin, upper arms, and abdomen, with minimal donor-site morbidity, given the widespread use of liposuction as a common cosmetic procedure. Notably, the combination of ADSCs with platelet-rich plasma has demonstrated the ability to stimulate the regeneration of periodontal ligament-like structures and alveolar bone in rats. These findings imply that ADSCs may hold promise for future clinical cell-based therapies in periodontal tissue engineering, representing a favorable cell candidate due to the ease of accessibility to human lipoaspirates and the low morbidity associated with their procurement [30,72].

### 5.3. Dental-Derived Mesenchymal Stem Cells for Periodontal Tissue Engineering

DMSCs encompass various types, including human dental pulp stem cells (DPSCs), stem cells from human exfoliated deciduous teeth (SHED), periodontal ligament stem cells (PDLSCs), stem cells from the apical papilla (SCAPs), gingival mesenchymal stem cells (GMSCs), dental follicle stem cells (DFSCs), tooth germ stem cells (TGSCs), and alveolar bone-derived mesenchymal stem cells (ABMSCs). The majority of these cells are derived from the neural crest and exhibit the potential for multipotent differentiation. They can be extracted from diverse tissues, including oral components such as alveolar bone, dental pulp, periodontal ligament, dental follicle, apical papilla, oral mucosa, and gingiva. DSCs, obtainable from exfoliated deciduous teeth or discarded dental tissues, present substantial potential as an alternative cellular source for tissue engineering for treating periodontitis and reducing systemic biomarkers [73,74,75]. Several types of DSCs can produce significant immunoinhibitory cytokines, such as TGF-β and IL-10. Additionally, DSCs can generate and release various bioactive factors that promote the growth and regeneration of multiple tissues, which are also related to machine learning [76,77,78,79].

## 6. Conclusions and Future Perspectives

Biomaterials employed for periodontal tissue regeneration consist of inorganic substances, polymeric materials, and composites. Inorganic biomaterials, possessing similar compositional and mechanical properties, are utilized for regenerating bone and cementum, while polymeric biomaterials are specifically chosen for periodontal ligament (PDL) regeneration. Most biomaterials used in periodontal regeneration are conventional types such as hydroxyapatite and beta-tricalcium phosphate (β-TCP). Despite exhibiting certain compositional similarities, these biomaterials do not fully replicate the intricate structures found in natural periodontal tissues.

It is imperative to develop novel, bio-inspired materials designed to closely replicate the architectural features of periodontal tissues at both micro- and nanoscale levels in order to achieve effective periodontal tissue regeneration. Regardless of all the challenges, the field of periodontal tissue regeneration is both exhilarating and experiencing rapid growth. The advancements within this field hold significant promise for enhancing the health of dental patients in the future. Subsequent investigations are essential to validate optimal concentrations of growth factors leading to the most effective regenerative results. Moreover, a more detailed understanding of the impacts of growth factors on the diverse components of periodontal support, implementing both soft and hard tissues, is necessary. Additionally, future investigations should delve into the effectiveness of tissue engineering strategies incorporating combinations of biologics. Finally, technologies for oral tissue engineering share a collective objective, which is to offer personalized treatment options for patients that optimize both functionality and esthetics and the overall quality of patient care.

## Figures and Tables

**Table 1:** Classification of barrier membranes.

**Nonresorbable**		**Resorbable**
Titanium mesh Titanium reinforced PTFE High-density polytetrafluoroethylene (d-PTFE) Expanded polytetrafluoroethylene (e-PTFE)		Polymeric-derived Collagen-derived
**First Generation**	**Second Generation**	**Third Generation**
Titanium mesh Titanium-reinforced e-PTFE Cellulose acetate High-density PTFE Expanded e-PTFE	Synthetic Natural	Barrier membranes with antimicrobial activity Barrier membranes with growth factors Barrier membranes with bioactive calcium phosphate incorporation

**Table 2:** Biomaterials used for periodontal tissue regeneration.

Biomaterials	Target Tissue	Characteristics
Hydroxyapatite	Alveolar bone and cementum	Osteoinductive and slow degradation
Tricalcium phosphate	Alveolar bone and cementum	Osteoinductive and bioabsorbable
Bioactive glass	Alveolar bone and cementum	Promote angiogenesis, osteogenesis, and antibacterial activity
Collagen	Periodontal ligament	Biocompatible and low mechanical characteristics
Chitosan	Alveolar bone, cementum and PDL	Biocompatible and antibacterial activity
Composite biomaterials	Alveolar bone	Support GTR membrane and promote bone regeneration

**Table 3:** Growth factors used for bone tissue engineering.

	Fibroblast Growth Factor	Platelet-Derived Growth Factor	Insulin-like Growth Factor	Transforming Growth Factor Beta	Vascular Endothelial Growth Factor	Bone Morphogenic Proteins
	FGF	PDGF	IGF	TGF-β	VEGF	BMP
**Mechanism**	Neo-vascularization, Cellular multiplication	Cell growth	Osteogenic differentiation	Osteogenic, chondrogenic differentiation	Neo-vascularization	Osteogenesis
**Functions**	Stimulating neo-vascularization by enhancing the osteoblast proliferation	Controller of the wound healing process	Inducing the proliferation of osteoblasts, bone matrix synthesis, activation of osteoclasts	Stimulating osteoprogenitor cell migration and regulating proliferation, differentiation, matrix synthesis	Controlling the activity of the endothelial cells and creating new blood vessels	Possess osteoinductive capabilities and stimulate bone formation

## Data Availability

Data are available from corresponding authors upon reasonable request.

## Abbreviations

DSCs	Dental stem cells
GTR	Guided tissue regeneration
GBR	Guided bone regeneration
PTR	Periodontal tissue regeneration
PDL	Periodontal ligament
TNF	Tumor necrosis factor
RANK	Receptor of nuclear factor-kappa
OPG	Osteoprotegerin
e-PTFE	Expanded Polytetrafluoroethylene
PGA	Polyglycolic acid
PLA	Polylactic acid
GFs	Growth factors
FGFs	Fibroblast growth factor
VEGFs	Vascular endothelial growth factor
IGFs	Insulin-like growth factor
TGF	Transforming growth factor
PDGFs	Platelet-derived growth factors
BMPs	Bone morphogenic proteins
GEM	Growth factor enhanced matrix
MSCs	Mesenchymal stem cells
GDF	Growth and differentiation factor
DPSCs	Dental pulp stem cells
SHED	Stem cells from human exfoliated deciduous
PDLSCs	Periodontal ligament stem cells
DFPCs	Dental follicle precursor cells
SCAP	Stem cells from apical papilla
BMSCs	Bone-marrow-derived MSCs
GFP	Green fluorescent protein
ADSCs	Adipose-derived stem cells
TGSCs	Tooth germ stem cells
